# Translational Assessments of Reward Responsiveness in the Marmoset

**DOI:** 10.1093/ijnp/pyaa090

**Published:** 2020-12-06

**Authors:** Lisa M Wooldridge, Jack Bergman, Diego A Pizzagalli, Brian D Kangas

**Affiliations:** 1 McLean Hospital, Belmont, Massachusetts, USA; 2 Harvard Medical School, Boston, Massachusetts, USA

**Keywords:** Animal model, anhedonia, reverse translation, touchscreen, ketamine, phencyclidine, marmoset

## Abstract

**Background:**

Anhedonia, the loss of pleasure in previously rewarding activities, is a prominent feature of major depressive disorder and often resistant to first-line antidepressant treatment. A paucity of translatable cross-species tasks to assess subdomains of anhedonia, including reward learning, presents a major obstacle to the development of effective therapeutics. One assay of reward learning characterized by orderly behavioral and pharmacological findings in both humans and rats is the probabilistic reward task. In this computerized task, subjects make discriminations across numerous trials in which correct responses to one alternative are rewarded more often (rich) than correct responses to the other (lean). Healthy control subjects reliably develop a response bias to the rich alternative. However, participants with major depressive disorder as well as rats exposed to chronic stress typically exhibit a blunted response bias.

**Methods:**

The present studies validated a touchscreen-based probabilistic reward task for the marmoset, a small nonhuman primate with considerable translational value. First, probabilistic reinforcement contingencies were parametrically examined. Next, the effects of ketamine (1.0–10.0 mg/kg), a US Food and Drug Administration-approved rapid-acting antidepressant, and phencyclidine (0.01–0.1 mg/kg), a pharmacologically similar *N*-methyl-D-aspartate receptor antagonist with no known antidepressant efficacy, were evaluated.

**Results:**

Increases in the asymmetry of rich:lean probabilistic contingencies produced orderly increases in response bias. Consistent with their respective clinical profiles, ketamine but not phencyclidine produced dose-related increases in response bias at doses that did not reduce task discriminability.

**Conclusions:**

Collectively, these findings confirm task and pharmacological sensitivity in the marmoset, which may be useful in developing medications to counter anhedonia across neuropsychiatric disorders.

**Significance Statement**

Anhedonia, the loss of pleasure from previously rewarding activities, is a prominent feature in several neuropsychiatric conditions, including major depressive disorder. Despite its transdiagnostic relevance, there are no effective therapeutics available to treat anhedonia. One major obstacle to medications development has been inconsistencies between preclinical measures of reward responsiveness across species. However, one laboratory-based task that has proven effective in objectively quantifying reward responsiveness in both laboratory animals and patients with mood disorders or anhedonia is the probabilistic reward task (PRT). The present studies empirically validated a touchscreen-based PRT for the marmoset, a small nonhuman primate with considerable translational value for preclinical neuropsychopharmacological research. These studies also documented the ability of the fast-acting antidepressant ketamine to selectively increase reward responsiveness in this task. Taken together, the present findings indicate the marmoset PRT might be useful in medications development for anhedonia.

## Introduction

Anhedonia, the loss of pleasure in, or lack of reactivity to, previously rewarding activities is a prominent feature and diagnostic criterion of major depressive disorder (MDD; American Psychiatric Association, 2013). Importantly, patients self-reporting higher levels of anhedonia are more likely to be treatment resistant (Spijker et al., 2001; Uher et al., 2012; Gabbay et al., 2015) and have a higher suicide risk (Xie et al., 2014; Ballard et al., 2017; Bonanni et al., 2019). Unfortunately, first-line antidepressants are often ineffective at treating anhedonia (Calabrese et al., 2014), further highlighting the urgent need for novel treatment strategies to restore positive mood in anhedonic patients.

A paucity of proven translational models presents a major obstacle in the preclinical development of improved antidepressants. This is due, in part, to functional differences between tasks used with clinical populations and laboratory animals (Der-Avakian et al., 2016). Most clinical tests for anhedonia rely on qualitative self-assessment questionnaires, which are dependent on verbal communication and therefore impossible to conduct in animals. Conversely, preclinical research tools to assess anhedonia in animals (reviewed in Scheggi et al., 2018) are often impractical to employ with human participants. Moreover, when functional animal task analogs have been examined in humans (e.g., preference for sweetened solutions), null findings have emerged in patients with MDD (Berlin et al., 1998; Dichter et al., 2010).

**Figure 1.**
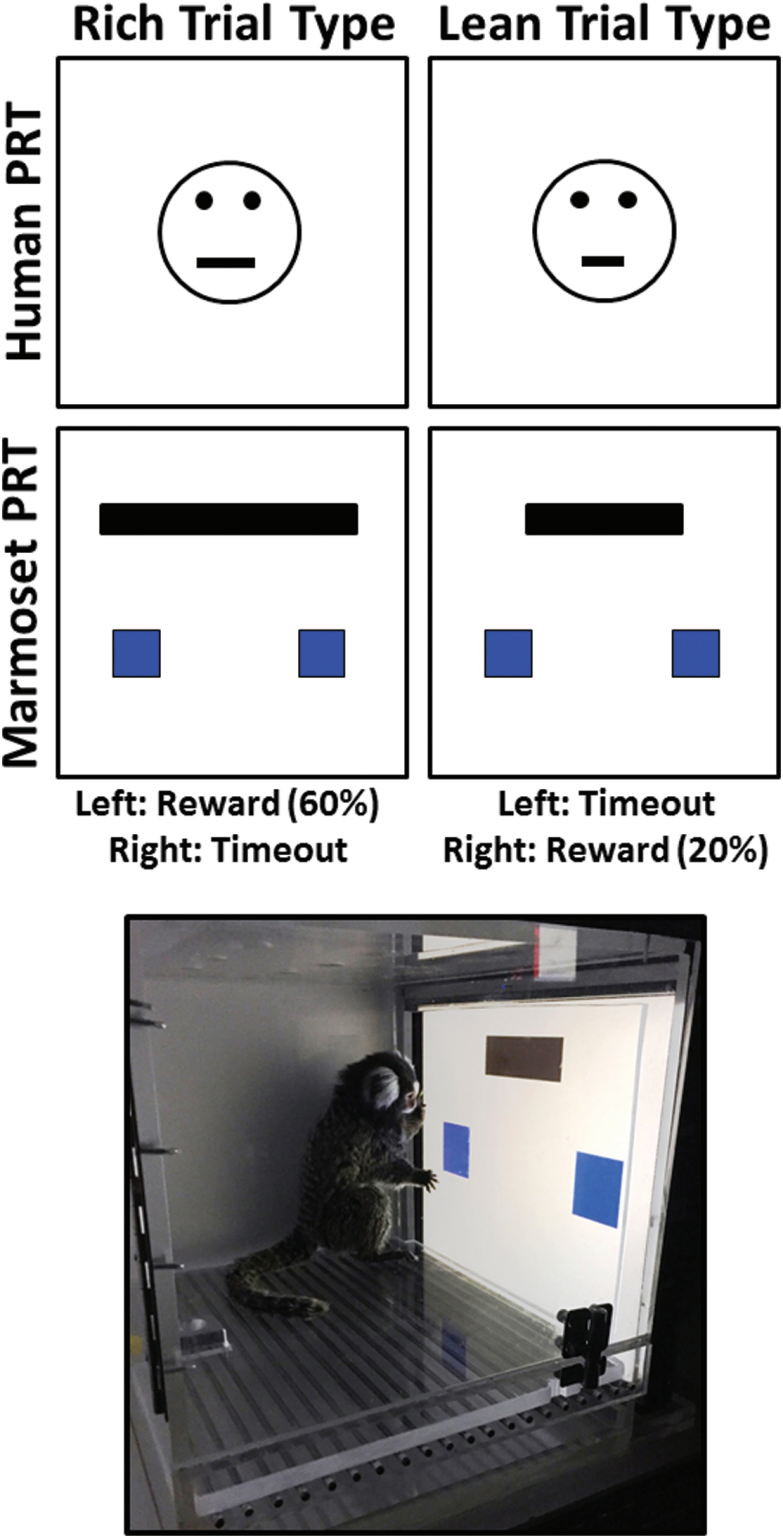
Task schematic for human PRT (top), marmoset PRT (middle), and photograph of marmoset responding (bottom).

The probabilistic reward task (PRT), a laboratory procedure designed for both human participants and laboratory animals (Pizzagalli et al., 2005; modified after Tripp and Alsop, 1999), provides a quantitative measure of reward learning (i.e., ability to modulate behavior as a function of reinforcement history). Originally developed as an objective tool to characterize reward deficit profiles in MDD and other mood disorders, the PRT uses discrimination methodology to quantify responsiveness to changes in reinforcer frequency. In the prototypical computerized task, human participants are instructed to discriminate between 2 briefly presented mouths that vary minimally in length on a cartoon face (Figure 1, top). Unbeknownst to participants, probabilistic contingencies are arranged so that correct responses on 1 alternative are rewarded 3 times more often (e.g., long line: rich alternative) than correct responses on the other alternative (e.g., short line: lean alternative). As predicted by signal detection theory (McCarthy and Davison, 1979; Macmillan et al., 1991; McCarthy et al., 1991), healthy control participants consistently develop a response bias in favor of the rich alternative and do so without disruption in overall task discriminability (Pizzagalli et al., 2005, 2008). However, participants with anhedonia typically exhibit a lower response bias than do healthy controls (Pizzagalli et al., 2005; Vrieze et al., 2013; Fletcher et al., 2015). Critically, blunted reward learning has been repeatedly documented to correlate with current and predict future anhedonia across multiple studies (Vrieze et al., 2013; Boger et al., 2014; Pizzagalli et al., 2009, 2014; Fletcher et al., 2015; Janes et al., 2015; Peechatka et al., 2015). It is important to note that although reward responsiveness generally, and the PRT specifically, does not capture the full spectrum of anhedonia, which is itself heterogeneous (Treadway and Zald, 2011; Pizzagalli, 2014; Rizvi et al., 2016), the PRT is a recommended assay to probe positive valence systems in the latest revision of the Research Domain Criteria (RDoC) matrix (NIMH, 2016).

The PRT has been reverse translated and functional task analogs have been empirically validated for rats using auditory stimuli (Der-Avakian et al., 2013, 2017) and, more recently, touchscreen-based line length stimuli to closely approximate the human task (Kangas et al., 2020). In the present studies, we adapted the touchscreen-based PRT for use in the marmoset, a small nonhuman primate that shares cortical and behavioral features with humans that are absent in rodents (Kishi et al., 2014). Of particular relevance to the present investigations is the ability to study prefrontal cortical activity in this nonhuman primate species. Compared with rodents, marmosets and other nonhuman primates share greater prefrontal cortical structural, functional, and genetic homology with humans (Mashiko et al., 2012; Oikonomidis et al., 2017; Roberts and Clarke, 2019). This is especially important to consider when modeling anhedonia and depression, as the ventromedial division of the prefrontal cortex shows hyperactivity and decreased functional connectivity with the ventral striatum and midbrain dopamine nuclei, and the severity of both findings correlates with anhedonic severity in depressed patients (Keedwell et al., 2005; Young et al., 2016; Borsini et al., 2020). Indeed, recent work in marmosets has shown that pharmacological activation of the subgenual anterior cingulate cortex (sgACC), a region of the ventromedial division of the prefrontal cortex corresponding to Brodmann’s area 25, by either blocking glutamate reuptake with dihydrokainic acid or enhancing glutamate release with CGP52432/LY341495, blunts conditioned anticipatory arousal to food reward, reflecting altered reward processing (Alexander et al., 2019). Marmosets, like humans but unlike rodents, also rely heavily on vision to navigate and communicate, and thus their visual cortex is much more similar to that of humans with respect to organization and functional connectivity, including with the inferotemporal and prefrontal cortex (Mitchell and Leopold, 2015). In addition, marmosets exhibit a number of social behaviors relevant to modeling depression that is, marmosets pair-raise their young, engage in cooperative behaviors with non-relatives, and utilize complex visual and auditory social communication (Miller et al., 2016). In addition, recent work has highlighted the suitability of this species for modeling parental deprivation- and social isolation-induced depression (Dettling et al., 2007; Galvão-Coelho et al., 2017). Notably, advances in precision gene editing techniques (e.g., CRISPR-Cas9) have facilitated the creation of a number of transgenic marmoset lines that, given the marmoset’s comparatively high rate of reproduction among primate species, have provided increasingly valuable neuroscience models (Sasaki et al., 2009; Okano et al., 2016; Kumita et al., 2019). In sum, the ability to complement cutting-edge translational tools now available to investigate the genetic and neural correlates of depression with a reverse-translational assay of reward learning in this nonhuman primate species will allow a more thorough investigation of the pathophysiology of depression and especially anhedonia.

In the current study, the sensitivity of the marmoset to probabilistic reinforcement contingencies was first assessed by systematically varying the asymmetry of rich:lean reward (experiment 1). Next, we evaluated task sensitivity to drug treatment following administration of ketamine, a recently US Food and Drug Administration-approved, fast-acting antidepressant (Kim et al., 2019), and phencyclidine, a pharmacologically similar *N*-methyl-D-aspartate (NMDA) receptor antagonist without known antidepressant efficacy (experiment 2).

## METHODS

### Subjects

Four adult male common marmosets (*Callithrix jacchus*) were individually housed in a climate-controlled vivarium with a 12-hourlight/dark cycle (lights on at 7 am). Subjects were maintained at approximate free-feeding weights via post-session portions of LabDiet New World Primate Chow (St. Louis, MO) and ZuPreem Marmoset Diet (Shawnee, KS). Fresh fruit, egg whites, mealworms, and environmental enrichment were provided daily. Subjects had unrestricted access to water in their home cage. The protocol was approved by the Institutional Animal Care and Use Committee at McLean Hospital in accordance with guidelines from the Committee on Care and Use of Laboratory Animals of the Institute of Laboratory Animals Resources, Commission on Life Sciences (National Research Council, 2011).

### Apparatus

Details and schematics of the marmoset touch-sensitive experimental chamber have been described previously (Kangas et al., 2016; Kangas and Bergman, 2017). Briefly, a Plexiglas chamber (25 × 30 × 35 cm) was situated in a sound- and light-attenuating enclosure (40 × 60 × 45 cm). A 17-inch touch-sensitive screen (1739L, ELO TouchSystems, Menlo Park, CA) comprised the inside right-hand wall of the chamber. An infusion pump (PHM-100–5, Med Associates, St. Albans, VT) outside the enclosure was used to deliver sweetened condensed milk solution into the shallow reservoir (diameter: 3 cm) of a plastic receptacle that was mounted 2 cm above the floor bars and centered on the left-hand inside wall. A speaker bar (NQ576AT, Hewlett-Packard, Palo Alto, CA) mounted above the touchscreen emitted auditory feedback. Experimental events and data collection were programmed in E-Prime Professional 2.0 (Psychology Software Tools, Inc., Sharpsburg, PA).

### Procedure

#### Line Length Discrimination Training

Marmosets were trained to discriminate between a short and a long line as described previously in rats (Kangas et al., 2020). Trials began with the presentation of a black line either 31.5 × 6.5 cm (long line) or 10.5 × 6.5 cm (short line). The line length trial type varied in a quasi-random manner across 100-trial sessions such that there were exactly 50 trials of each length, but a given length would not be presented more than 5 times in a row. Subjects were trained to respond on 1 of two 5  × 5 cm blue virtual response boxes, presented left and right of center 5 cm below the line stimulus, depending on the length of the line (Figure 1, middle). Whether the left or right response box was assigned to the long or short line length was counter-balanced across subjects. A correct response was reinforced with the presentation of a yellow screen, a 440-Hz tone, and 0.15 mL of 30% sweetened condensed milk, simultaneously delivered over 880 ms, and was followed by a 5-second blackout period. An incorrect response immediately resulted in a 10-second blackout period. A correction procedure in which each incorrect trial was repeated until a correct response was made (Kangas and Branch, 2008) was implemented during initial discrimination training and was discontinued after <10 repeats of each trial type occurred per session. Discrimination training sessions continued without correction until accuracies for both line length trial types were ≥80% correct for 3 consecutive sessions.

### Experiment 1: Parametric Assessment of Asymmetry in Probabilistic Schedules

To examine the effects of asymmetrical probabilistic contingencies on PRT performance, subjects were exposed to a 4-day probabilistic testing protocol each week as follows. On Monday, all correct responses during the session were rewarded (1:1 [100%:100%]). For 2 of 4 subjects, the line length to be associated with the rich and lean contingency for the remainder of the week was determined during this training session by designating the line length with a higher mean accuracy as the lean alternative. For the other 2 subjects, the line length with a higher mean accuracy was the stimulus designated as the rich alternative. During test sessions conducted on Tuesday through Thursday, subjects were exposed to a 3:1 (60%:20%) rich:lean probabilistic schedule of reward in accord with the human task protocol. Thus, 60% of correct responses to 1 of the line lengths (rich alternative) and 20% of correct responses to the other line length (lean alternative) were reinforced. Thereafter, subjects were exposed each week to the 4-day probabilistic testing protocol using schedules of reinforcement of 4:1 (80%:20%), 1:1 (20%:20%), and 2:1 (40% : 20%) rich:lean probabilistic contingencies in that order.

### Experiment 2: Effects of Ketamine and Phencyclidine on PRT Performance

To investigate the effects of drug treatment on PRT performance, subjects were exposed to a 4-day acute drug testing protocol each week as follows. Training sessions in which all correct responses were rewarded (1:1 [100%:100%]) were conducted on day 1. The 3:1 (60%:20%) rich:lean probabilistic contingencies were programmed during days 2–4, and the effects of pre-session drug administration on PRT performance were examined during the day 4 session. Saline, ketamine (1.0, 3.2, 10.0 mg/kg), or phencyclidine (0.01, 0.032, 0.1 mg/kg) was administered 2 hours prior to the session no more than once per week. The 2-hour pretreatment interval and ketamine dose range was based on previous studies of NMDA receptor antagonists in traditional animal models of depression (e.g., Autry et al., 2011; Sarkar and Kabbaj, 2016; Zhao et al., 2020) and to ensure acute motor-impairing effects of drug administration were no longer present during the touchscreen task. The 7-day washout period between drug tests was based on previous human studies documenting ketamine’s ability to produce reductions in self-reported anhedonia for a maximum of 3 days (Lally et al., 2014, 2015). Ketamine was of primary interest in the present studies and, therefore, was examined in all subjects first. Phencyclidine was examined in all subjects next, following a washout period of at least 6 weeks, during which subjects underwent intermittent training sessions designed to maintain discriminative performance. All subjects experienced all doses of each drug in a mixed order as detailed in Table 1.

**Table 1.** Timeline of Drug Tests (mg/kg) Among Subjects

	Ketamine				Washout	Phencyclidine		
Subject	Week 5	Week 6	Week 7	Week 8	Weeks 9–14	Week 15	Week 16	Week 17
1	1.0	3.2	10.0	Saline		0.032	0.1	0.01
2	3.2	1.0	10.0	Saline		0.1	0.032	0.01
3	10.0	3.2	1.0	Saline		0.032	0.1	0.01
4	10.0	1.0	3.2	Saline		0.1	0.01	0.032

### Data Analysis

The implementation of probabilistic contingencies yields 2 primary dependent measures: response bias and task discriminability, which can be quantified using equations derived from signal detection theory by examining the number of correct and incorrect responses for rich and lean trial types. Specifically, response bias was calculated using the following log *b* equation:

$$\begin{array}{l} \log b=0.5*\log\left(\frac{\left(Rich_{Correct}+0.5\right)*\left(Lean_{Incorrect}+0.5\right)}{\left(Rich_{Incorrect}+0.5\right)*\left(Lean_{Correct}+0.5\right)}\right). \end{array}$$ (1)

High bias values are produced by high numbers of correct responses for rich trials and incorrect responses for lean trials. Discriminability was calculated using the following log *d* equation:

$$\begin{array}{l} \log d=0.5*\log\left(\frac{\left(Rich_{Correct}+0.5\right)*\left(Lean_{Correct}+0.5\right)}{\left(Rich_{Incorrect}+0.5\right)*\left(Lean_{Incorrect}+0.5\right)}\right) \end{array}$$ (2)

High discriminability values are produced by high numbers of correct responses for both rich and lean trials. (0.5 is added to all parameters in both Eq. 1 and Eq. 2 to avoid instances where no errors are made on a given trial type, thus making log transforms impossible.) The utility of these equations has been repeatedly confirmed in prior human studies (Pizzagalli et al., 2008; Vrieze et al., 2013; Boger et al., 2014; Fletcher et al., 2015; Janes et al., 2015; Peechatka et al., 2015) and rodent studies (Der-Avakian et al., 2013; Lamontagne et al., 2018; Kangas et al., 2020). Importantly, although log *b* values serve as a principal datum of reward responsiveness, its modification, for example via drug treatment, is particularly meaningful when log *d* values are not perturbed. That is, it is critical to confirm that increases in response biases are not simply a byproduct of deficits in task discriminability but rather represent an increased response allocation toward the richly rewarded stimulus at the expense of lessened response allocation toward the lean stimulus (which explains why robust shift in response bias can occur in the context of no changes in overall discriminability). Other PRT performance outcomes, including accuracy (percent correct) and reaction time (latency from line presentation to response), were calculated and presented as session-wide group means (± SEM) for rich and lean trials. All data (log *b*, log *d*, accuracy, reaction time) were analyzed using repeated-measures ANOVA. For accuracy and reaction time, the repeated-measures factor trial type (rich vs lean) was added to the model. When appropriate, ANOVAs were followed by post-hoc tests for linear, quadratic, or cubic trends/contrast to evaluate the statistical significance of increasing asymmetry of rich:lean probabilities and dose-response functions, and Bonferroni’s tests to evaluate the statistical significance of rich and lean trial type on accuracy and reaction time as well as the statistical significance of drug doses compared with saline treatment. The criterion for significance was set at *P *< .05. All statistical analyses were conducted using GraphPad Prism 8 Software (San Diego, CA).

### Drugs

Ketamine hydrochloride and phencyclidine hydrochloride were obtained from Sigma-Aldrich (St. Louis, MO). Both drugs were dissolved in 0.9% saline solution and were administered via intramuscular injection in volumes of 0.3 mL or less. Drug doses (1.0, 3.2, 10.0 mg/kg ketamine; 0.01, 0.032, 0.1 mg/kg phencyclidine) are expressed in terms of their free base weights.

## RESULTS

**Figure 2.**
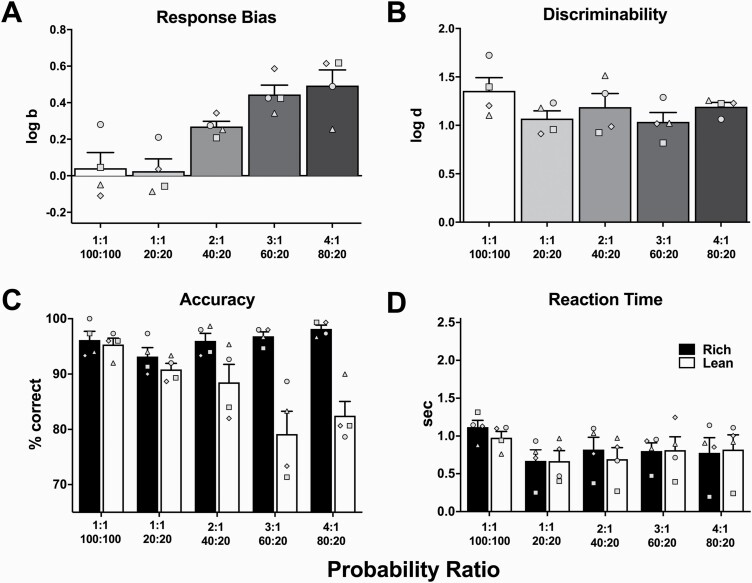
Effects of rich:lean probabilistic contingencies on (A) log *b*, (B) log *d*, (C) accuracy, and (D) reaction time. Bars represent group mean (±SEM) of 3-day averages for each condition. Data points represent values for individual subjects. *n *=* *4.

All subjects learned the line-length discrimination to ≥80% correct criterion following an average of 26.25 (range: 20–33) training sessions, each of which was completed in approximately 30 minutes. The effects of manipulating the asymmetry of rich:lean probabilistic contingencies on task performance are presented in Figure 2. When there was an equal probability of reinforcement for correct responses on rich and lean stimuli (1:1 conditions), a near-zero response bias (log *b*) was observed regardless of whether all trials were reinforced (100%:100%) or only 20% of each trial type was reinforced (20%:20%). Exposure to 2:1 (40%:20%), 3:1 (60%:20%), and 4:1 (80%:20%) probabilistic contingencies produced log *b* values that increased systematically with increases in the asymmetry of the reinforcement schedule (Figure 2A). The relationship between increases in asymmetry of rich:lean probability and the increase in log *b* value was significant (*F*[1.85, 5.54] = 18.18, *P *=* *.004), as was the linear trend (*P < *.0001). Critically, alterations in response bias were not accompanied by significant changes in discriminability (log *d*; Figure 2B; [*F*{1.96, 5.88} = 1.01, *P* = .42]). Taken together, these data indicate that manipulating probabilistic contingencies systematically alters response bias without altering task discriminability.

This conclusion was reinforced when examining accuracy scores. Specifically, and as shown in Figure 2C, accuracy on rich trials was consistently higher than lean trials (*F*[1,6] = 20.89, *P *=* *.004). Increasing asymmetry between probabilities of reinforcement produced an upward pattern in accuracy on rich trial types (*F*[1.50, 4.49]* *=* *3.46, *P* = .13). This effect was accompanied by a downward trend in accuracy on lean trial types across probabilistic contingencies (*F*[1.36, 4.07]* *=* *6.76, *P *=* *.06). These PRT accuracy outcomes illustrate how log *b* values can increase systematically with increases in the asymmetry of probabilistic reinforcement schedules (Figure 2A) despite the fact that log *d* values were similar across probabilistic conditions (Figure 2B). That is, increased asymmetry increases biased performance toward the rich stimulus, which produces higher accuracy during that trial type and, during the same test session, lower accuracy during the lean trial type, resulting in similar log *d* values across probabilistic conditions. The interaction between probability of reinforcement and trial type was significant (*F*[4,24]* *=* *8.98, *P* =* *.001). As shown in Figure 2D, there was no difference in reaction time between rich and lean trials (*F*[1,6]* *=* *0.07, *P *= .81), nor was there a significant difference in reaction times between probabilistic contingencies (*F*[1.82, 10.95]* *=* *3.41, *P *=* *.07).

**Figure 3.**
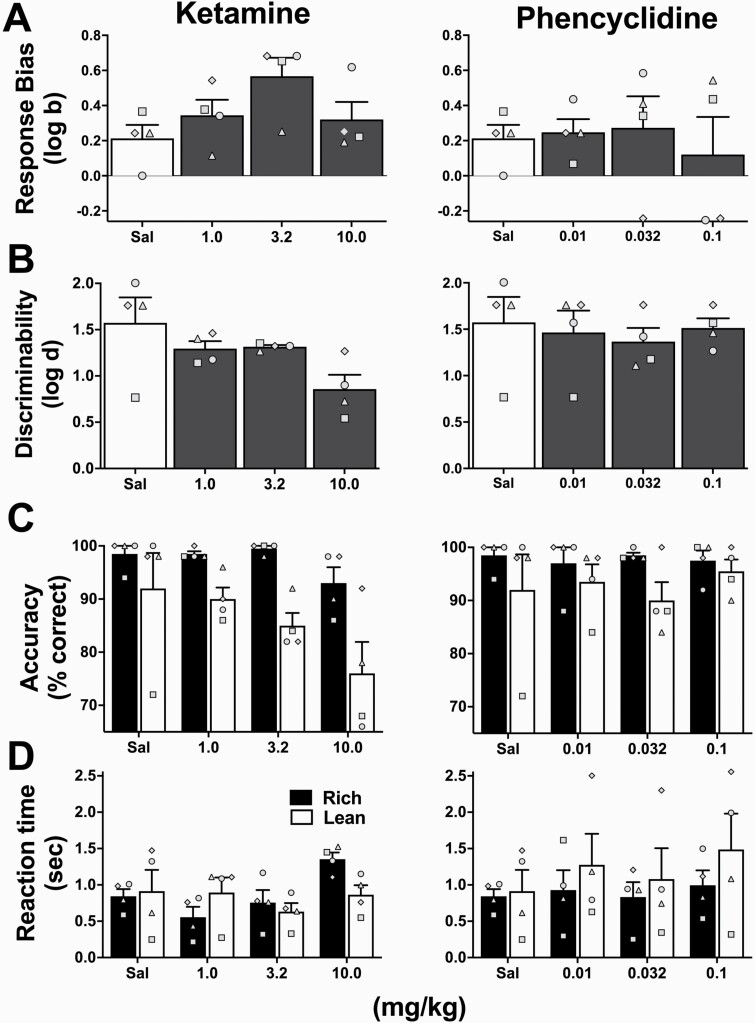
Effects of ketamine (left), and phencyclidine (right) saline (Sal; data repeated in left and right panels), on (A) log *b*, (B) log *d*, (C) accuracy, and (D) reaction time. Bars represent group mean (±SEM). Data points represent values for individual subjects. *n *=* *4.

The effects of pretreatment with saline, ketamine (1.0, 3.2, 10.0 mg/kg), and phencyclidine (0.01, 0.032, 0.1 mg/kg) on PRT performance are presented in Figure 3. Pretreatment with ketamine produced an “inverted-U” dose response function of log *b* values relative to saline (Figure 3A), with the dose of 3.2 mg/kg ketamine producing a maximal increase in log *b* values in all 4 subjects. Consistent with this observation, the cubic effect was significant (F[1,3]* *=* *16.72, *P* = .02). Ketamine pretreatment did not significantly alter log *d* compared with saline (*F*[1.50, 4.50]* *=* *4.49, *P *=* *.09; Figure 3B); however, pretreatment with 10.0 mg/kg ketamine produced a small decrease in discriminability. As in experiment 1, accuracy on rich trials was significantly higher than on lean trials (*F*[1,6]* *=* *11.80, *P *=* *.01) and there was also a significant effect of drug on accuracy (*F*[2.01,12.04]* *=* *5.06, *P *=* *.03; Figure 3C). While 1.0 mg/kg and 3.2 mg/kg produced an increase in rich trial accuracy accompanied by a small decrease in lean trial accuracy compared with saline, 10.0 mg/kg produced a small decrease in both rich and lean trial accuracies. Ketamine treatment produced a significant effect on reaction time (*F*[2.18,13.07]* *=* *5.36; *P *=* *.02), largely driven by a significant decrease in reaction time relative to saline following administration of 1 mg/kg (*P *=* *.03; Figure 3D).

Relative to saline, pretreatment with a range of phencyclidine doses did not produce significant alterations in log *b* (*F*(1.99,5.98]* *=* *0.21, *P *=* *.82; Figure 3A) or log *d* (*F*[1.33,3.99]* *=* *0.29, *P* = .68; Figure 3B). Likewise, there was no effect of phencyclidine on accuracy (*F*[1.63,9.76]* *=* *0.22, *P* = .76; Figure 3C) or reaction time (*F*[1.66,9.98]* *=* *2.25, *P *=* *.16; Figure 3D). A higher dose of 0.32 mg/kg phencyclidine produced untoward behavioral effects in the first subject tested, precluding further assessment in additional subjects.

## Discussion

The present studies empirically validated a touchscreen-based PRT to assay reward learning in the marmoset. Increasing the asymmetry of rich:lean probabilistic contingencies produced systematic increases in response bias, verifying the sensitivity of marmoset performance under these task conditions and extending similar patterns recently described in rats (Kangas et al., 2020). In addition, optimized task conditions for drug testing yielded log *b* values (approximately 0.2–0.3) that are highly similar to those in previous studies with healthy control human participants (e.g., Pizzagalli et al., 2005, 2008) and rats (e.g., Der-Avakian et al., 2013, 2017; Lamontagne et al., 2018; Kangas et al., 2020). Furthermore, pretreatment with ketamine, but not phencyclidine, significantly increased response bias for the rich stimulus. These findings are in agreement with previous work using other animal models of reward processing. For example, Stuart et al. (2015) documented ketamine’s ability to attenuate previously acquired negative affective biases in rats using a task of bowl-digging for reward. Likewise, Hales et al. (2017) showed that ketamine, but not phencyclidine, produced antidepressant-like effects in rats using a judgment bias task for low vs high food reward magnitudes. In recent studies of marmosets by Alexander et al. (2019), deficits in reward processing were induced by over-activation of the sgACC, which has been documented previously to be over-active in depressed patients (Drevets et al., 2008; Keedwell et al., 2009). Following these manipulations, ketamine was able to reverse blunted anticipatory arousal to a conditioned stimulus associated with high-incentive food reward (Alexander et al., 2019).

Severity of anhedonia in MDD patients, including that associated with reward learning, predicts poor treatment outcome with conventional antidepressants (Nutt et al., 2007; Uher et al., 2012). Additionally, first-line antidepressants such as selective serotonin reuptake inhibitors themselves blunt emotional reactivity in some patients, producing symptomology that can resemble or exacerbate depression-induced anhedonia (McCabe et al., 2010). In contrast, accumulating evidence suggests that ketamine may be effective for patients with treatment-resistant depression, and a formulation has recently obtained US Food and Drug Administration approval for this purpose (Kim et al., 2019). Ketamine has also been shown to produce reductions in self-reported anhedonia in patients with treatment-resistant bipolar disorder (Lally et al., 2014) and MDD (Lally et al., 2015) for up to 3 days following infusion. In keeping with these findings, Alexander et al. (2019) found that while ketamine blunts the anticipatory arousal brought on by stimulation of the marmoset sgACC, the selective serotonin reuptake inhibitor citalopram does not.

Despite its clinical success, ketamine’s antidepressant mechanisms of action are not well understood. While ketamine is known to be an NMDA receptor antagonist that interacts with the receptor at the PCP binding site, other NMDA antagonists that bind to the same site do not necessarily exhibit antidepressant effects (Newport et al., 2015; Zanos and Gould, 2018). Consistent with such reports, the present studies confirm that phencyclidine, another ligand at the same binding site, did not produce changes in log *b* and was largely behaviorally silent at doses up to those with nonspecific behaviorally disruptive effects. Differences in activity at the NMDA receptor or at other receptors may account for the dissimilar effects of ketamine and phencyclidine in the present studies. For example, the presence of physiological concentrations of magnesium in patch clamp electrophysiology experiments impedes the ability of phencyclidine (Lerma et al., 1991), but not ketamine (Liu et al., 2001; Gideons et al., 2014), to antagonize NMDA receptor function, suggesting that ketamine acts more robustly than phencyclidine during periods of low neuronal activity. Alternatively, the activity of the ketamine metabolite (2R,6R)-hydroxynorketamine at AMPA receptors, independent of NMDA receptor antagonism by ketamine itself, may account for its antidepressant effects (Zanos et al., 2016; Lumsden et al., 2019). Regardless of ketamine’s mechanism of action, it is interesting that, even in the absence of manipulations to decrease reward responsiveness, response bias could be increased in all subjects by treatment with a drug known to have fast-acting antidepressant efficacy. This is consistent with previous studies in unstressed rats documenting the ability of d-amphetamine and scopolamine to produce dose-related increases in log *b* (Kangas et al., 2020). It is noteworthy that studies in marmosets examining ketamine’s effects on anticipatory arousal have documented selective effects following the smaller dose of 0.5 mg/kg during conditions of sgACC overactivation (Alexander et al., 2019). Future marmoset research capitalizing on the touchscreen-based PRT will be needed to examine the extent to which response bias (log *b*) is blunted by programmed stressors such as parental separation (Dettling et al., 2007), social isolation (Galvão-Coelho et al., 2017), or sgACC overactivation (Alexander et al., 2019); for such studies, it will be important to conduct full-dose response functions to determine the lowest doses of ketamine that can rescue reward responsivity.

A few caveats regarding the present studies warrant consideration. First, only male marmosets were examined. Diagnoses of mood disorders, including MDD, are more prevalent in women (Hyde and Mezulis, 2020), and, for future preclinical drug development, it will be important to determine whether this task will also yield orderly findings in female marmosets. Second, although phencyclidine did not produce any systematic alterations in response bias or other PRT performance outcomes, the testing order of ketamine, saline, and phencyclidine was not counterbalanced across subjects. Third, given clinical reports of ketamine’s enduring effects on behavior and mood, and studies in marmosets showing ameliorative effects 7 days after ketamine treatment (Alexander et al., 2019), future studies using these procedures could arrange longer periods between ketamine administration to allow for characterization of its time course. Fourth, although it was important to arrange an extended washout period between the ketamine and phencyclidine tests to avoid possible carry-over effects, intermittent touchscreen training sessions were conducted to maintain PRT performance during the 6-week washout period, which resulted in somewhat higher accuracy of the lean trial types during the phencyclidine tests. Because the PRT relies on stimulus ambiguity in signal detection to produce a response bias via probabilistic contingencies (McCarthy, 1983), it is possible that the task may have been less sensitive during the assessment of phencyclidine’s effects than during experiments with ketamine. More generally, an upward drift in task accuracy via extended experimental exposure needs to be considered when evaluating PRT outcomes, especially in long-term studies commonly conducted in nonhuman primate species. Nevertheless, the results indicate that, at minimum, phencyclidine does not produce robust alteration to response bias in the PRT, which is consistent with its lack of known antidepressant efficacy.

Taken together, these results empirically validate a touchscreen-based PRT for marmosets and confirm its pharmacological sensitivity via ketamine’s ability to alter reward responsiveness. As discussed above, although anhedonia is a multifaceted construct, reactivity to reward and reward learning are important RDoC positive valence systems subdomains implicated in anhedonic phenotypes. Recent developments in precision gene editing have highlighted the marmoset as a nonhuman primate laboratory animal of considerable interest and translational value. Future studies using the touchscreen-based PRT to compare performance of wild-type marmosets and transgenic mutants could accelerate drug development efforts for depression and other mood disorders in which anhedonic phenotypes and deficits in reward responsiveness are prominent.

## Statement of Interest

Over the past 3 years, Dr Pizzagalli has received consulting fees from BlackThorn Therapeutics, Boehreinger Ingelheim, Compass Pathway, Engrail Pharmaceuticals, Otsuka Pharmaceuticals, and Takeda Pharmaceuticals; one honorarium from Alkermes; stock options from BlackThorn Therapeutics; and funding from NIMH, Brain and Behavior Research Foundation, the Dana Foundation, and Millennium Pharmaceuticals. Dr Pizzagalli has a financial interest in BlackThorn Therapeutics, which has licensed the copyright to the human version of the Probabilistic Reward Task through Harvard University. Dr Pizzagalli’s interests were reviewed and are managed by McLean Hospital and Partners HealthCare in accordance with their conflict of interest policies. All other authors report no financial relationships with commercial interests.
